# The paradox of helping: Contradictory effects of scaffolding people with aphasia to communicate

**DOI:** 10.1371/journal.pone.0180708

**Published:** 2017-08-14

**Authors:** Alex Gillespie, Julie Hald

**Affiliations:** Department of Psychological and Behavioural Science, London School of Economics and Political Science, London, United Kingdom; University of Kent, UNITED KINGDOM

## Abstract

When interacting with people with aphasia, communication partners use a range of subtle strategies to scaffold, or facilitate, expression and comprehension. The present article analyses the unintended effects of these ostensibly helpful acts. Twenty people with aphasia and their main communication partners (*n* = 40) living in the UK were video recorded engaging in a joint task. Three analyses reveal that: (1) scaffolding is widespread and mostly effective, (2) the conversations are dominated by communication partners, and (3) people with aphasia both request and resist help. We propose that scaffolding is inherently paradoxical because it has contradictory effects. While helping facilitates performing an action, and is thus enabling, it simultaneously implies an inability to perform the action independently, and thus it can simultaneously mark the recipient as disabled. Data are in British English.

## Introduction

‘The gift not yet repaid debases the man who accepted it … Charity wounds him who receives, and our whole moral effort is directed toward suppressing the unconscious harmful patronage of the rich almoner.’

([1]: p.63)

Helping is a social activity that must be analysed both in terms of the activity being helped and the meaning of receiving help. Providing help aims to enable the recipient to perform an action; but, simultaneously, helping indexes assumptions about the ability of the recipient, the authority of the helper, and their relationship. Most of the literature on helping has focused on facilitating activities. A smaller literature has shown that people often resist receiving help–echoing Mauss’ statement, in the above quotation, that any form of gift creates an imbalance that wounds one’s identity. The present article combines these literatures, arguing that helping entails a very specific communicative entanglement in which the practical and relational effects of helping are contradictory; while helping can empower, it can also simultaneously index disempowerment.

We analyse conversations between persons with aphasia (PAs) and familiar communication partners (CPs). The helping behaviour is scaffolding communication, for example, reading aloud, breaking down sentences, speaking for, prompting, and facilitative gestures. Our first analysis describes the scaffolding that is provided, showing that it is most frequently done by CPs and usually effective. Our second analysis reveals an unintended effect of scaffolding, namely, positioning CPs as dominant. The third analysis reveals PAs alternating between requesting and resisting scaffolding. To understand why PAs simultaneously request and resist scaffolding, we introduce the concept of the paradox of helping.

### Aphasia within informal relationships

Aphasia is a communication disability that can be caused by stroke, brain injury, brain tumours, infection or a progressive neurological condition. In terms of communication, aphasia usually impairs a person’s ability to speak, write, read, and understand speech. In terms of identity, the potential outcomes of dependency upon others, unemployment, and social isolation [2] can result in a diminished sense of self [3].

Close social relationships are an important resource in adapting to aphasia [4]. However, these relationships can also be undermined by aphasia [3]. First, becoming an informal caregiver is a challenging transition, entailing a vulnerability to physical and psychological strain [5]. Second, maintaining these social relationships becomes more difficult due to the communication impairment itself [6].

Given the importance of close relationships in adapting to aphasia, a lot of research has focused on enhancing communication within these relationships [7]. For example, PAs can receive training to maximise use of retained capacities [8] and video feedback can be used to enhance communication within relationships [9]. Also, taking advantage of the relational nature of communication, some interventions have focused on communication partner training [10–11]. The evidence indicates that training communication partners to facilitate the PAs expression and reception is effective [12–13], with a general correlation between communication strategies and communication success [14]. However, communication partner training can have diverse outcomes [15], and efficacy depends upon the extent and type of language deficits. Accordingly, any training in facilitating communication needs to be individually tailored to the particularities of the PAs aphasia [16–17].

### Scaffolding communication

‘Scaffolding’ is a metaphor initially used in child development research to describe how an adult or expert can provide cognitive support to enable ‘a child or novice to solve a problem, carry out a task or achieve a goal’ just beyond their ‘unassisted efforts’ ([18]: p.90). The concept of scaffolding is also evident in Vygotsky’s [19] observation that children are better at performing tasks when receiving support from an expert who guides attention, augments memory, and structures the task [20].

Stone [21] identifies four aspects to scaffolding. First, scaffolding involves the recruitment of a learner to typically valued activity. Second, the expert’s support is not fixed but titrated in response to the learner’s changing capabilities over time. Third, strategies of scaffolding can vary widely given their contingency to whatever should arise. Fourth, support is gradually removed resulting in a transfer of responsibility from expert to learner.

The concept of scaffolding has proved useful beyond child development [22], being applied to adults who have learning disabilities [21], dementia [23], cognitive impairment [24], and aphasia [25]. The concept of scaffolding, in these domains, focuses attention on the way in which interaction partners provide help that is relational, temporary, and contextually calibrated [4]. However, the idea of removing the scaffolding, enabling independent task performance, is often inapplicable when conditions are chronic or deteriorating.

Research on aphasia has identified a broad range of scaffolding strategies used by communication partners, including: Checking agreement, repetition and demonstrating understanding [10]; speaking-for, steering the interaction and correcting [26]; reformulating, offering options, praising and using assistive technology [27]; controlling the task or environment and giving simple directives [28]; third-turn repair [29]; responding to problematic talk [30]; reading aloud and spelling-out [31]; prompting [32]; and pointing and gesturing [33]. Despite the preponderance of scaffolding strategies that have been identified, there has been little systematic examination of the extent to which PAs use these strategies, the efficacy of the strategies, what effects they have on dominance within the relationship, and the extent to which these strategies are requested or resisted.

## Overview of research: Examining the effects of scaffolding

We take a sociocultural approach [34] that assumes a perspectival social world [35]. Meaning arises from the situated and historical interaction of different perspectives [36–37]. Emergent meanings are only partially shared [38]; each action or utterance within the social interaction affords multiple, often unexpected, interpretations in the social world [39–40]. Accordingly, our aim is to examine not only the practical efficacy of scaffolding communication, but also its unintended effects.

Our methodological strategy has been to draw freely on any qualitative and quantitative techniques that can advance the analysis. While we have found conversation analysis procedures useful in conceptualising and transcribing the interactions, we are not intending to report a conversation analysis study. Specifically, while we agree that it is problematic to speculate about motivations within an interaction sequence, we nevertheless assume (in line with our sociocultural approach) that motivations do exist and that they are an important component of the concept of helping.

The data we analyse are problem-solving conversations between PAs and their main CPs. Three analyses are reported, examining, in turn, the effects of scaffolding on enabling communication, on dominance, and on the relationship.

Analysis 1 asks: What scaffolding strategies are evident, who uses them, and are they effective? Scaffolding research has focused almost exclusively on ‘the expert,’ glossing over the ‘the learner’s’ capacity for agency [21]. PAs are active in scaffolding, using gestures [27, 33] and assistive technology [41]. Moreover, PAs try to guide CP scaffolding, requesting CPs to repeat, slow down, wait, or read aloud [10, 42]. A second limitation of the scaffolding literature, that we address, is the assumption that scaffolding is effective when evidence is emerging to suggest that it is not always effective [43].

Analysis 2 asks: What effects does scaffolding have on dominance within conversations? While the literature has generally been enthusiastic about the potential of CP scaffolding, one study has observed that CPs tend to dominate conversations, holding the floor, and acting as primary speaker [44], while PAs have trouble making initiations [45].

Analysis 3 asks: Why do people with aphasia both resist and request scaffolding? Extensive literature demonstrates a widespread reluctance to receive help [46]: People with depression fear stigmatization [47]; people in organisations worry about appearing incompetent [48]; and children in school want to avoid looking dumb [49]. In relation to aphasia, CPs’ attempts at speaking-for are often resisted [26]. Our third analysis explores the idea of a tension between the potential of scaffolding to simultaneously facilitate communication and make salient disability.

## Methodology

Twenty-four PA-CP dyads were recruited through a Speech and Language Therapy Service within the NHS. An NHS Research Ethics Committee reviewed the project, procedures, and documents and approved the study (07/S0501/73). Participation was voluntary and no remuneration provided. Participants provided written consent for participating in the study, being video recorded, and allowing us to use the verbal and visual data in publications. Four dyads dropped out for health reasons. Among the 20 dyads that completed participation, the average number of months since onset was 30 and the aetiology was stroke (*n* = 18) and traumatic brain injury (*n* = 2). Speech and language therapists rated participants’ aphasia as: 5 mild, 2 mild/moderate, 6 moderate, and 7 severe; 11 were mainly expressive and 9 expressive and receptive. Fifteen PAs had concomitant hemiplegia and/or dyspraxia. The PAs (11 male, 9 female) had an average age of 59. The CPs (13 female, 7 male) were partners (*n* = 16), adult children (*n* = 2), and parents (*n* = 2). All dyads were living together.

**Fig 1:**
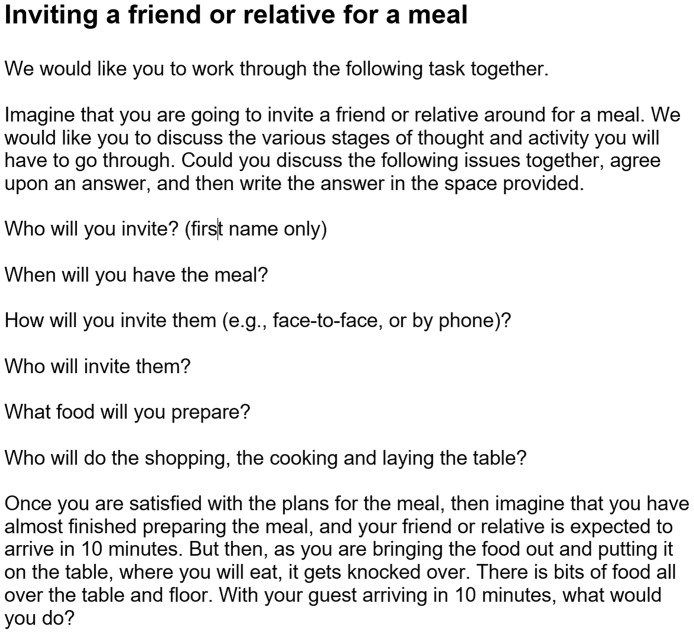
The joint task.

Piloting with unstructured conversation showed dyads falling into routine and non-challenging exchanges that were non-comparable. Accordingly, we developed an artificial task designed to be challenging enough to elicit scaffolding and equally unfamiliar to all dyads. We created the ‘inviting a friend or relative for a meal’ task as a culturally-familiar activity judged appropriate for the population. The task (Fig 1) was introduced as a “joint activity.” There was one A4 sheet with the task and participants were asked to write answers directly onto the sheet.

The task was conducted during home visits by a speech and language therapist, with a first visit explaining the study and consent procedure, and a second visit to gather the data. Home visits ensured the talk elicited occurred in a natural setting even if it would not have occurred without the researchers’ intervention [50].

All 20 conversations were video recorded. The mean duration was 7:02 minutes (range 2:19 minutes to 16:29 minutes). Verbal data (2 hours 34 minutes) were transcribed using the conversation analytic conventions set out by Gail Jefferson [51] with nonverbal data added where salient.

**Fig 2:**
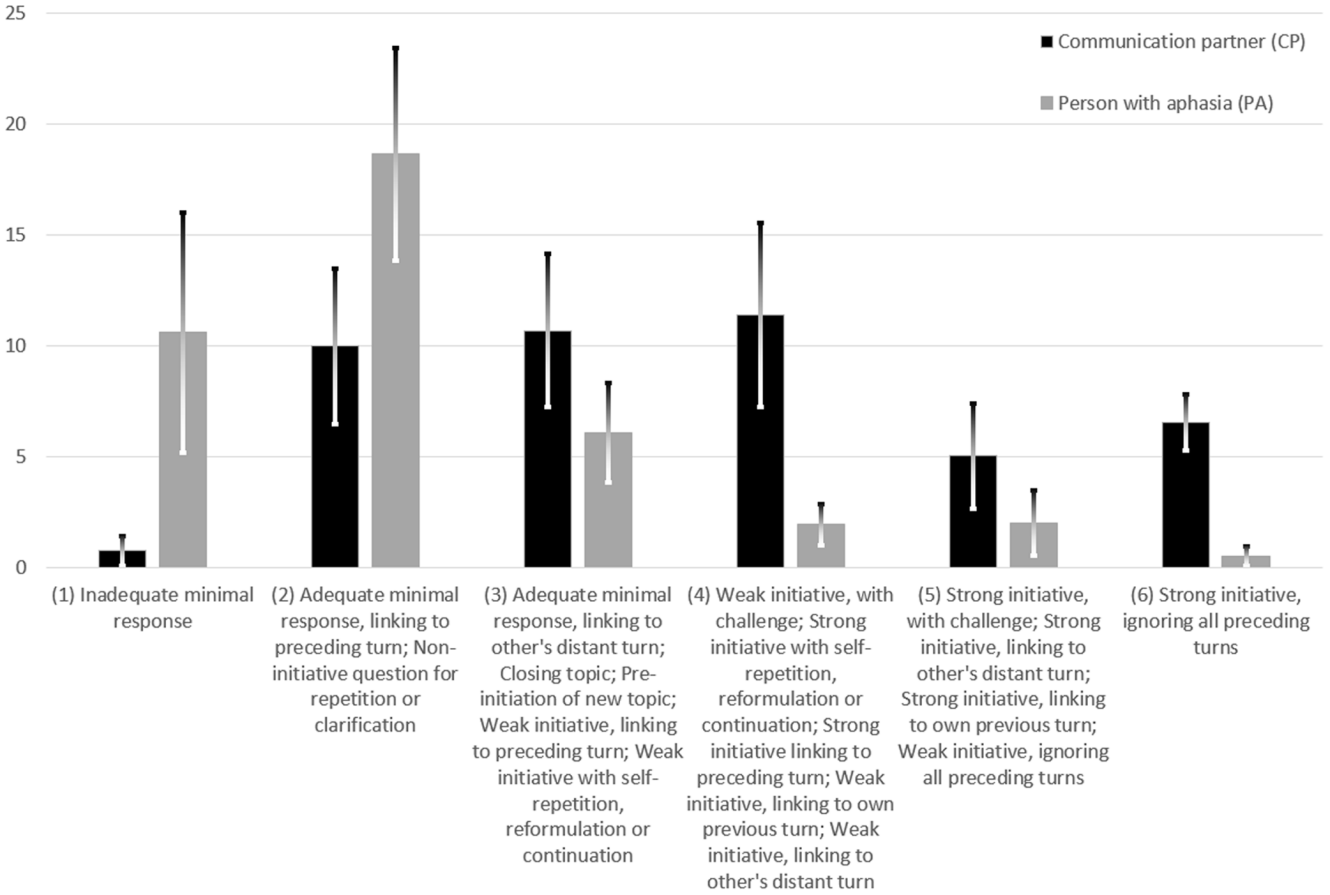
Mean frequency of utterance strength (with standard deviations).

Three analyses were conducted corresponding to the three research questions. The first analysis began trying to code the data according to the scaffolding strategies identified in the section ‘Scaffolding Communication.’ These were then refined to have clearer textual operationalization and minimal overlap (see Table 1 for coding categories). Scaffolding efficacy was assessed, where possible, by examining whether it was accepted by the recipient and/or whether it resulted in a communication accepted by the initiator. The second analysis followed the procedure of Initiation-Response Analysis [52] (see Fig 2 for coding categories). The third analysis coded instances of both requesting help and resisting help, along with the more general indicators of resistance, namely, interrupting and disagreeing (see Analysis 3 for coding categories). Three excerpts, selected because they had multiple illustrations of the coding categories, are presented and analysed. This final analysis then used Peirce’s [53] ideas about paradoxes to interpret the observed tension between requesting and resisting help.

**Table 1:** Usage and efficacy of scaffolding strategies.

Scaffolding strategy	CP (*n*)	PA (*n*)	CP/PA^†^ (%)	CP %^††^ Efficacy	PA % Efficacy
**Checking agreement**: often a question, sometimes reformulating or other-repetition	79	1	99/1	84	100
**Speaking-for**: completing or 'filling out' the interlocutor's inadequate adjacent turn	40	1	98/2	85	100
**Reformulating**: modifications of original that reduce complexity, increase redundancy, or chunk elements	164	11	94/6	57	91
**Praising**: any positive verbal feedback, such as 'that's it good' or 'right'	16	1	94/6	50	100
**Repair initiations**: utterances treating as problematic a second turn misunderstanding of a first turn	89	12	88/12	67	67
**Offering options**: any suggestion of single or multiple possible solutions to the task questions	147	25	85/15	70	92
**Correcting**: utterances that correct an aspect of the interlocutor's preceding utterance or action	12	2	86/14	50	0
**Steering**: moving to the next question or changing topic forgoing any mutually agreed transition	228	57	80/20	68	84
**Reading aloud**: loud-reading without amendment to the original task text	120	32	79/21	59	81
**Prompting**: seeking to elicit a response, e.g. 'who' and 'when' questions, excluding offering options	165	50	78/22	64	90
**Sounding-out**: loud letter-by-letter spelling out of problematic lexical items	22	7	76/24	73	86
**Directing**: directives that issue an order, e.g., 'just read it from the beginning'	16	9	64/36	56	89
**Other-repetition**: unmodified repetition of an adjacent lexical item or clause	38	35	52/48	*	*
**Demonstrating understanding**: engagement with adjacent turn, often prefaced with ‘so’	43	58	43/57	*	*
**Assistive technology**: using an artifact for communication, e.g., writing-out, using symbol cards	9	12	43/57	67	50
**Gesturing**: nonverbal communication e.g., pointing, nodding, head-shaking, shrugging, and tracing	122	215	36/64	*	*

^†^ Percentage of all instances of the scaffolding type performed by CP vs PA

^††^ Percentage of instances of the scaffolding type that were effective

* Efficacy could not be assessed because the scaffolding has indeterminate responses.

To assess reliability, 20% of the data was double coded by an independent MSc graduate. Applying a Cohen’s kappa, the interrater agreement was found to be moderate for the analysis of scaffolding strategies (*k* = 0.527), repair initiations (*k* = 0.580), Initiation-Response Analysis (*k* = 0.508), and instances of resisting and requesting help (*k* = 0.458). Coding of repair initiations was done separately to other scaffolding strategies because it involved a greater unit of analysis (three turns-at-talk).

## Analysis 1: Usage and efficacy of scaffolding strategies

Table 1 shows that CPs and PAs engaged in scaffolding 1,310 (*M* = 65.5, range 29 to 149, *SD* = 33.08) and 528 (*M* = 26.4, range 7 to 67, *SD* = 16.2) times respectively. Table 1 collapses CPs and PAs into groups, and it is important to note that there was considerable variability in how frequently the strategies were used between dyads (see data in S1 File). Specifically, while most CP used all strategies (although 3 CP never read aloud, 2 CPs never initiated a repair, and 2 CPs never gestured) many PAs did not use particular strategies (17 PAs never used an assistive technology and 13 PAs never read aloud, however, only 1 PA did not gesture). This variability likely reflects the nature and extent of the aphasia [16], participants’ attitudes towards strategies [54], the particularities of the dyad’s relationship and trial and error experience with the strategies. We will consider the strategies used by CPs and PAs in turn.

### Scaffolding by communication partners (CPs)

Starting with the CPs, it is important to note that few strategies were used by all CPs. This likely indicates that CPs, on the basis of their familiarity with the PAs, were making selective use of strategies [17, 54]. The most common scaffolding strategies used were steering, reformulating, prompting, offering options, gesturing, reading aloud, repairing, and checking agreement. Less frequently, there were also episodes of teacher-like correcting and praising. We will consider these strategies in turn.

‘Steering’ refers to attempts to ensure the activity’s optimal progression. These turns would often use phrases such as “right,” “alright” and “so then” as the CP took control of the task and moved the dyad from one question to the next. CPs’ control of the progression through the task compares to how dominant parties in institutional settings (e.g., doctors, interviewers, judges) utilise their greater allowance of discursive resources to direct the other’s contributions [55]. Such control implies a lower status for the PA [56].

‘Reformulating’ entailed simplification either in real time (i.e., whilst reading aloud) or ex post facto as part of a repair. Reformulation was dominated by CPs (93.71%) and had poor efficacy (56.71%) relative to the other efficacy scores. While some substantial chunking of communication was evident, the majority of instances entailed minimal changes to the syntactic structure of the task questions. For example, CPs tended to exchange the impersonal nouns and deictic references with more proximal ones. While apparently well-intended and occasionally even an expression of solidarity (e.g., “you” for “we") [57], such minimal reformulation can imply that the PA’s marker of trouble (e.g., “um”) had been a product of inattentiveness or even idleness [58].

‘Prompting’ usually entailed CPs asking questions about “who,” “when,” “what” and “how” to solicit answers. Problematically, the person who does the prompting significantly determines the way in which an interaction can sensibly proceed [59–60]. CPs tended to prompt to help the PA arrive at a solution without providing it directly. Their implicit assumption might have been that their role was not to provide answers but, instead, to facilitate the PA to provide the answers.

‘Offering options’ entailed introducing ideas which the other presumably would or could not have summoned alone. Most frequently, the options offered were names of people to invite, days when the meal could be scheduled, and possible dishes (e.g., “chicken?” and “what about stir-fry?”). At the outset, a distinction must be drawn between those scaffolds which consisted of two or more options and those which consisted of one. The latter stood for 89.32% of effective offerings. While such single options give less room for PAs to exercise choice, these yes/no interrogatives enabled PAs to express their position with “yes” to close topics and “no” to keep topics open [61]. Offering multiple options was more challenging given it required PAs repeat one of the options named. PAs would regularly agree instead (e.g., “yes”), forcing the CP to offer each option again singly. As with reading aloud and sounding-out, offering either single or multiple options, especially when they failed and had to be repeated, made salient the communication difficulty.

‘Gesturing’ was most prevalent among PAs (see below), but, when used by CPs it was usually to unobtrusively augment communication whilst speaking. CPs, for example, would gesture while reading “bringing the food out,” “putting it on the table” and it getting “knocked over.” Due to this augmentative function of gesturing it was not possible to evaluate efficacy; with the benefit that it likely improved communication without ever resulting in overt failure.

‘Reading aloud’ refers to any reading of the task-text verbatim (without reformulation) that was sufficiently loud to be communicative. Although widespread among CPs, it was relatively ineffective (59.17%), possibly due to the task-text’s low redundancy. CPs’ persistence likely sought to spare PAs struggling to read the text. Nevertheless, reading aloud can imply that the PA needed to be read aloud to, echoing the genre of asymmetrical interaction used with young children in classrooms [62].

‘Repair initiations’ were carried out to restore misunderstandings, usually following problematic demonstrations of understanding, repetitions or responses. While repairs usually follow misunderstandings [63], in our data they often followed non-understandings. A typical example is a CP offering two food options and the PA responding “yes,” leading the CP to reiterate the options more slowly. Having such non-understanding made salient risks positioning the PA as conversationally incompetent. Moreover, as reported elsewhere [64], the dyads often took more than three turns to re-establish mutual understanding, thus adding salience to the communication failure.

‘Checking agreement’ assessed whether participants’ partially shared intersubjectivity had endured the most recent sequence of turns. Although explicit checking is rare in typical conversation [65], it occurred relatively frequently in our data and was almost completely dominated by CPs. Arguably, by implication of doing the checking, this strategy consolidated CPs’ authoritative role within the task [66]. These yes/no questions were particularly effective (83.54%). However, it should be noted that agreement is not necessarily understanding [65]. In fact, agreeing is a robust way of feigning understanding when there is a desire to avoid embarrassment, support a respected interlocutor or close an unstimulating topic given that it requires no proof of understanding be provided [67].

CPs also dominated ‘speaking for,’ ‘praising,’ ‘correcting,’ and ‘directing.’ While each of these strategies was relatively infrequently used, together, they suggest a pattern of interaction that is very asymmetrical. Speaking-for [26], correcting [68], and directing [69] evidently claim an entitlement over the recipient. But, even paying compliments, especially between adults, can reproduce and solidify a perceived asymmetry in competence [70]. Phrases such as “look how you spelled fish” (correcting), “no, try again” (directing) and “right, well done” (praising) suggest a genre of interaction more closely associated with parent-child or teacher-child interactions than with adult-adult interaction.

### Scaffolding by persons with aphasia (PAs)

Turning to PAs it is important to note that collectively they used all scaffolding strategies, but, many were used very infrequently, and no strategy was used by all PAs. Again, this diversity underscores the fact that strategies are selected and tailored according to the particularities of the aphasia, relationship, and history of interaction [17]. The majority of their scaffolding was gesturing, demonstrating understanding, steering the interaction, and prompting. Although PAs engaged in less scaffolding than CPs, their efficacy was higher (83.18% vs 66.21%), perhaps indicating a high degree of responsiveness of CPs to scaffolding attempts.

‘Gesturing’ accounted for 40.72% of all PA scaffolding. It was mainly used to supplement verbal utterances and facilitate CP comprehension of information and feelings nonverbally [71]. PAs’ gestures were adaptive and rich. For example, when the word ‘drink’ escaped a PA, he motioned putting a glass to his lips. Common gestures included pointing to the person who would perform a task or pointing to a word on the task sheet, as an indirect attempt to recruit help [72].

‘Demonstrating understanding’ refers to rephrasing of the preceding turn so as to demonstrate comprehension and, as such, is more robust than either checking agreement or other-repetition for establishing intersubjectivity. Overt demonstrations are exceedingly rare in ordinary conversation [73] and none were found in the data. Rather, there were what may be called ‘tacit claims of recognition’ ([73]: p.257). By virtue of their tacitness, these claims fulfilled the preference that intersubjectivity be verified without unnecessary interruption to a conversation’s progressivity [73]. Demonstrating understanding let PA independently close sequences and preface next-position matter by showing sufficient interest in the other’s preceding turn [74–75].

Although ‘steering’ was dominated by CPs (see above), 14 of the PAs also engaged in steering. These were often assertive interruptions, sometimes resistive attempts to claim control over some aspect of the task, often accompanied by taking the task sheet, and trying to read the questions or write an answer. For example, in one dyad the conversation began with the CP taking the task sheet saying “okay” (i.e., clearly steering the task), and then the PA saying “it says who will we invite,” thus making a claim to steer the task.

Similarly, although ‘prompting’ was dominated by CPs, it is also a strategy that 19 of the PA engaged in. However, while CPs tended to prompt to help the PA arrive at an answer without providing it directly, PAs tended to prompt to convey non-understanding or to check their understanding. Thus, for example, PAs would suggest answers (i.e., “chicken?”) or interpretations of the task-sheet (i.e., “go shopping?”) as tentative suggestions, almost checks of comprehension, and thus inviting correction. This recurring difference in how prompting was used bolstered participants’ epistemic asymmetry at the expense of the PA [66].

### Summary of the first analysis

The first analysis has three main findings. First, scaffolding is variable but two-sided; every strategy is used by at least some CPs and PAs. Second, scaffolding is generally effective. Third, CPs engage in scaffolding 2.48 times as frequently as PAs. These findings beg the question: Might CPs’ frequent scaffolding interventions, especially their use of strategies reminiscent of teaching young children and frequent steering, lead to asymmetrical domination within the conversations?

## Analysis 2: Interaction dominance

A simplistic way to assess communication dominance is to compare the number of words spoken by each party. Taking this approach to our data appears to show imbalance, with CPs speaking on average 412.4 words (range 186 words to 656 words; *SD* = 133.74 words) and PAs speaking on average 156.05 words (range 27 to 323; *SD* = 101.71 words). It also shows heterogeneity, as 5 PAs spoke less than 50 words and 7 spoke more than 200 (see data in S1 File). But, this overall imbalance could be a by-product of the fact that PAs often find speech production difficult. Accordingly, we focus on what Linell, Gustavsson and Juvonen [52] term ‘interactional dominance,’ that is, one person managing to direct and control the other party’s actions and utterances whilst also avoiding being directed and controlled.

Linell and colleagues [52] developed the Initiation-Response Analysis to assess interaction dominance, it entails assigning each turn in a verbal interaction to one of 18 mutually exclusive and exhaustive categories. Turns are distinguished in terms of responsiveness to previous turns, initiation of something new, strength or assertiveness, adequacy, scope, focality, and whether they link to the previous utterances of self or other. Each of the 18 codes is assigned a turn strength score on a 6-point ordinal scale, ranging from the weak and minimally responsive with no initiation (1 point) to non-responsive and strongly initiating (6 points).

Fig 2 shows the interaction dominance distribution, grouped by overall strength score, revealing that PAs were mainly responsive while CPs were making much more initiation. While the modal turn for PAs was the adequate minimal response, linking to the preceding turn (scored 2), for CPs the modal turn was a strong initiative, ignoring all preceding turns (scored 6).

**Fig 3:**
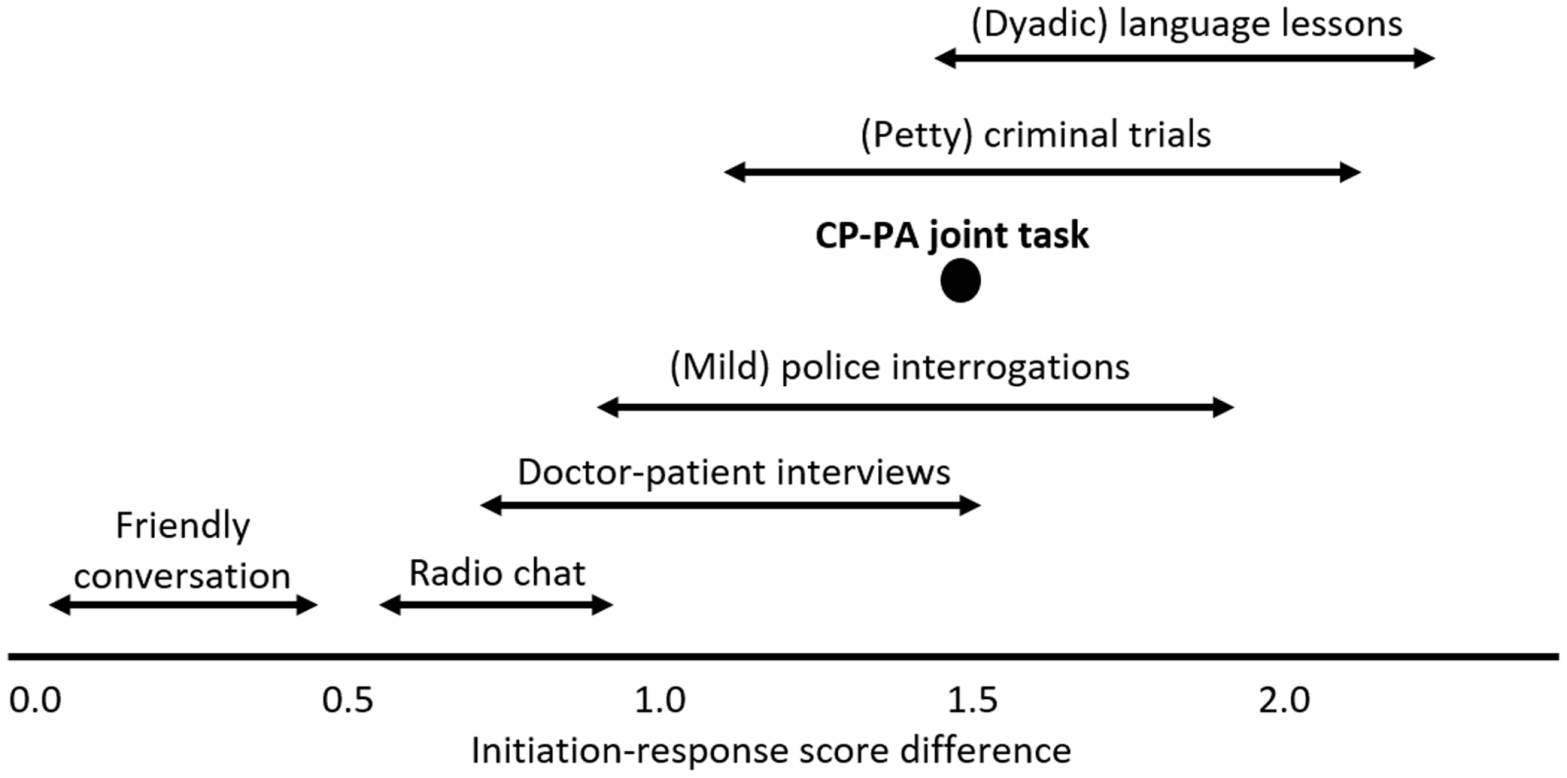
CP-PA initiation response difference compared to other interaction types (data from ([43]: p.433)).

The median utterance strength scores, termed the ‘IR index,’ for CPs was 3.58 (range 3 to 4.5 points, *SD* = 0.52) and for PAs was 2.08 (range 1 to 3 points, *SD* = 0.41). The difference between these IR indices, termed the ‘IR difference,’ was 1.5. Linell, Gustavsson and Juvonen ([52]: p.433) report IR differences for diverse interactions, ranging from informal conversation between friends (IR difference = 0.07) to a court trial (IR difference = 1.97). Fig 3 compares the IR difference that we found with some of their data.

To explore the source of the large dominance asymmetry we used the IR methodology to calculate coefficients for balance, obliqueness, solicitation, and fragmentation (Table 2). The balance-coefficient is the percentage of turns that responded focally to a preceding turn and which also contained some initiation. It indicates how conversation-like the dialogue was. The CP and PA balance-coefficients of 10.46% and 10.05% do suggest a good deal of balanced talk. Indeed, these balance-coefficients are comparable to those reported for other atypical dyads [76–77].

**Table 2:** Initiation-response indices and coefficients of interactional asymmetry.

	IR-index	Balance	Obliqueness	Solicitation	Fragmentation
Communication partner	3.58	10.46%	16.42%	38.58%	34.42%
Person with aphasia	2.08	10.05%	3.14%	3.14%	11.68%

The obliqueness-coefficient reveals non-cooperation between participants; it is the percentage of initiatives which ignored, challenged or commented upon their preceding turns. The obliqueness-coefficient for CPs was much higher than for PAs (16.42% vs 3.14%). This shows that CPs sometimes ignored or criticized their partners, partially accounting for the IR difference. An example of a weak initiative with a challenge, is after the PA tried to write “go to café for meal” and the CP said “well, that’s not ‘meal’ you’ve written there, it’s ‘menu’ you’ve written there.”

The solicitation-coefficient is the percentage of turns which demanded a response. Not only was soliciting common, but, CPs did it over ten times more frequently (38.58% vs. 3.14%), thus making a significant contribution to the IR difference. The CP utterances coded as strong initiations soliciting a response included: “Do you want me to phone them?” “Would you like to put it in some kind of sauce?” “Right, who are you inviting?” and “Right, what would you do?” Such instances of solicitation suggest that CPs oriented to the activity as one of trying to get their partner with aphasia to talk. Indeed, it is as if the CPs were siding with the task, and taking responsibility for administering the task to the PA.

Finally, the fragmentation-coefficient is the percentage of non-locally linked initiatives which did not pick up on an adjacent turn but which linked to an earlier one, therein weakening the dialogue’s coherent flow. Such turns constituted 34.42% of CP turns and just 11.68% of PA’s. That is, CPs often treated their partner’s contributions as off-topic or inadequate. This even occurred in some instances when the PA contributions were entirely on-topic. For example, one PA had just finished reading the first question, about who to invite, and said quickly and clearly “Alex.” The CP then said: “Right, who would you invite, first name only?” thus ignoring the PA’s turn.

Summarising the initiation-response analysis, we can say that although participants were asked to treat the task as a “joint activity,” many CPs positioned themselves as soliciting responses and coordinating the task. Their initiatives were not *asking* for information but trying to *elicit* a response that would demonstrate the PA’s understanding of the locally-relevant matter [77]. Indeed, when these initiating/responding roles reversed, CPs would usually try to turn the job of answering back to their partner. Most striking, however, is that despite soliciting responses, CPs sometimes ignored the responses they received or treated them as insufficient (indicated by the high fragmentation-coefficient).

## Analysis 3: Resisting and requesting help

The third analysis examined the effect of scaffolding on PAs. Specifically, how do PAs respond to both the effectiveness of scaffolding and the potential interaction domination that it can result in? We coded both explicit requests for help and explicit resisting help, along with more implicit indicators of resistance, namely interrupting and disagreeing. Table 3 summarises our findings, showing that although PAs often request help, they also frequently resist it.

**Table 3:** Indicators of request or resistance.

Indicator	CP (*n*)	PA (*n*)	CP/PA (%)
**Requesting help**: Explicit verbal or nonverbal request for help, excluded creating opportunities for help	1	44	2/98
**Resisting help**: Direct verbal or nonverbal refusal of help provided in the previous turn	14	36	28/72
**Disagreeing:** Explicit disagreement, does not include suggesting alternative	14	32	30/70
**Interrupting**: Overlapping speech, excluding nonverbal communication	82	133	38/62

‘Requesting help’ included any verbal or nonverbal solicitation of support. It was done almost exclusively by PAs (97.78%; 6 PAs never requested help). The one time a CP requested help was inadvertent; he asked himself how to spell “substitute” only to have his partner provide the correct spelling. The number of requests is low considering the frequency of scaffolding observed (45 vs. 1,838). This suggests a high degree of coordination in which scaffolding is often being provided (and accepted) without PA having to explicitly request it. While requesting help positions the PA in control of what scaffolding they receive and when, too many such requests would risk positioning them as dependant and incapable. Arguably, CPs are thus trying to pre-empt requests by responding to what Kendrick and Drew [72] term ‘projectable troubles.’

‘Resisting help’ included express refusals of help, hushing to silence it, and head shaking. Some resistance was subtler, for example one PA simply ignored her partner’s help in spelling “meal,” opting instead to write “food.” The majority of resisting help was done by PA (72%; 8 PAs never resisted help), but, taking account of how often scaffolding was offered (1,310 vs. 528 times) reveals that both PAs and CPs were equally likely to openly resist help when it was offered (2.72% vs 2.64%). However, focusing only on explicit resistance underestimates the extent of PA resistance which also manifested indirectly through disagreements and interruptions.

‘Disagreeing’ comprised of adjacently positioned action-opposition turns. The typical format of disagreeing-thru-agreement was often replaced with participants’ unconcealed orientation to ‘the prior turn as arguable’ ([78]: p.23). Whilst 71.43% of caregiver ‘disagreeing’ was modulated by, for example, the preface “well” [79], its interpolation by laugh particles [80], or weak modalization (e.g., “maybe” and “I think”) [81] to soften and work up its reluctance, only half of PAs did the same. That is to say, 50% of PAs’ disagreeing utterances were unapologetic and aggravated [78] for directly stating “no” or declaring the exact opposite. Thus, not only were PAs much more likely to disagree (69.57% vs 30.43%), but the way they disagreed was also much more vociferous and direct, arguably positioning themselves as independent. However, again, we found variability, as 5 PAs never disagreed.

**Fig 4:**
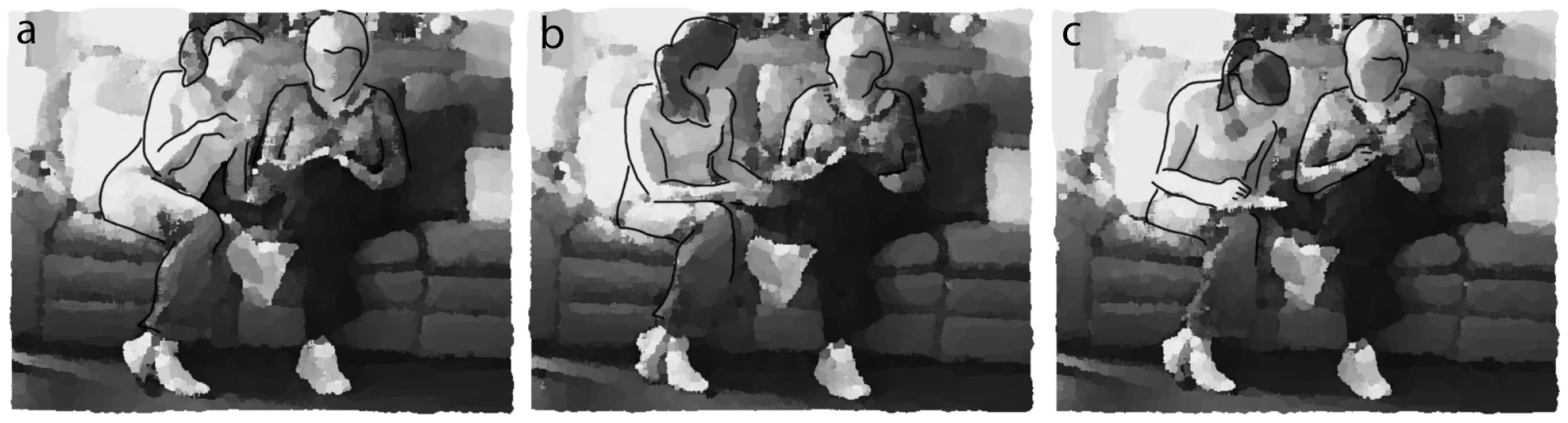
a-c. Anonymised video stills from excerpt 1 (CP is on left).

Excerpt 1 illustrates disagreeing. It begins after the mother with aphasia and her daughter have read the task question (“what food will you prepare?”). The daughter prompts her mother “what d’you reckon?” (line 01) and the mother tries to say “spaghetti bolognese” (lines 02–03). After some shared laughter about this, with the daughter being particularly animated, and leaning into her mother while looking at her (lines 03–04, Fig 4a), the daughter turns to the researcher to ask, in a joking manner “h(h)ave you set aside a lot of ti(h)me ↑for ↑her?”. The reason, she states, is because “w(h)e’re a special case” (lines 06–09). The use of “we” is ostensibly an attempt to share ownership over the problem she has just highlighted (i.e. taking extra time). However, these turns clearly draw attention to the mothers aphasia: it is directed at the researcher and pertains to the mother’s prior attempt to say “spaghetti.” The mother, then, with apparent concentration clearly enunciates the problematic phrase “Spaghetti> bolog↑ne(h)se!” (lines 11–12). The exclamatory quality as well as the rising intonation with which it is produced both suggest the mother’s relief at having clearly articulated the phrase. When her daughter then turns to her asking whether she would like to spell it out (line 15, Fig 4b), the mother blurts out “n(h)o!”. The daughter takes the sheet and begins to write while, in passing, suggesting that their guest might not like mince (lines 20–25). Throughout the preceding turns the mother’s impairment has been made salient. Now her judgement about what food to cook for her chosen guest is being called into question in front of the researcher. She openly disagrees with her daughter twice (lines 22–25, Fig 4c).

(1) Mother (PA) disagreeing with daughter (CP) (03:24)

01  CP  what d'you reckon?             Prompting

02  PA  em: (1.8) spa:: (1.2) I-I know that heh (0.9)

03     skabetti [heh heh heh! (Fig 4a)

04  CP  [heh heh! (Fig 4a)

05     (0.7)

06  CP  h(h)ave you ((looking at the researcher)) set

07     aside a lot of ti(h)me ↑for ↑her? (0.8)

08     w(h)e're a special case ↑h(h)ere w(h)e're heh

09     heh!

10     (1.2)

11  PA  <what food will you make? (0.5) Spaghetti>    Reading aloud

12     bolog↑ne(h)se! [heh heh heh

13  CP  [heh heh heh heh!

14     (0.3)

15  CP  d(h)o you want to write it? (Fig 4b)       Prompting

16  PA  n(h)o! [heh heh heh!

17  CP  [heh heh

18     ((CP takes sheet and writes answer))

19     (1.1)

20  CP  °I don't how much he likes mince mum°

21     (1.0)

22  PA  well he had it here befo:re (Fig 4c)        Disagreeing

23  CP  °no: I think he was being polite° [heh         Disagreeing

24  PA  [well                   Interrupting

25     ↓I::'ll speak to ↑him heh heh!          Disagreeing

26  CP  .hh right who will prepare it?          Steering

‘Interrupting’ was defined as the annexation of one person’s speaking rights by another [78, 82]. Interrupting may be affiliative or disaffiliative with the other’s stance [83], but always forces abandonment so as to fulfil the conversational norm of minimal overlap [84]. As with disagreement, interrupting often followed a sequence in which the PA was negatively positioned.

**Fig 5:**
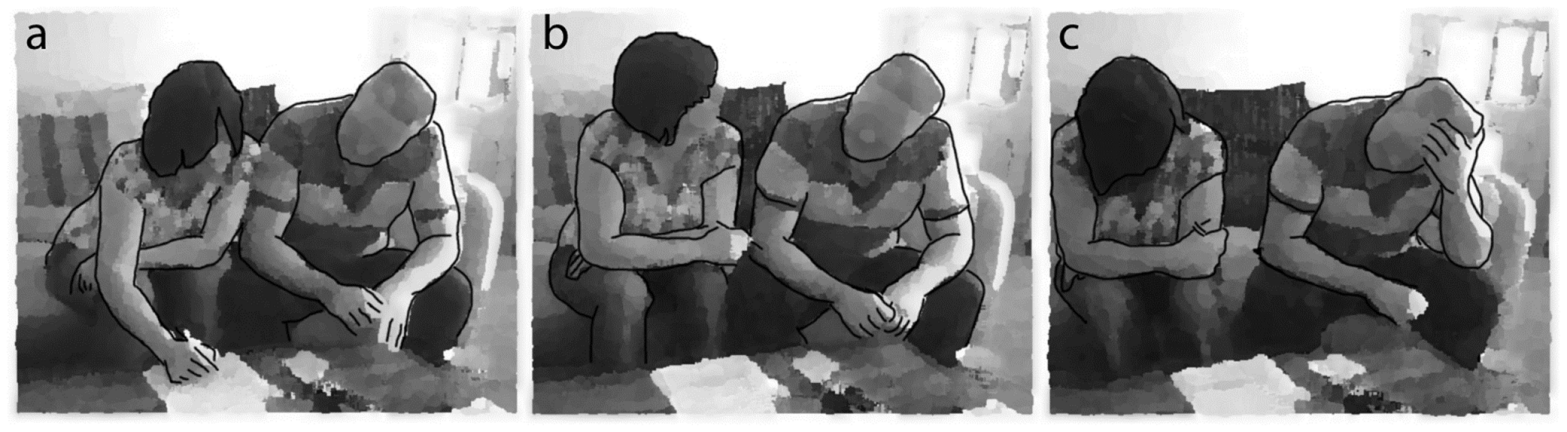
a-c. Anonymised video stills from excerpt 2 (CP is on left).

Excerpt 2 illustrates interrupting. It begins with a wife leaning into her husband and writing their agreed-upon answer that she would set the table (lines 01–02, Fig 5a). After writing, she turns to her husband, looking him in the eyes, and rationalises the agreement, telling her husband with aphasia he would “probably .hhh (0.6) forget” (lines 04–05, Fig 5b). The husband interrupts (line 06), suggesting she could also do the dishes. The CP abandons her previous turn and disagrees in a friendly way, but the PA interrupts, again, insisting that she will do the dishes. This creates some nervous laughter, the bodies briefly separate, and the husband rubs his head (lines 11–13, Fig 5c). Arguably, the husband’s interruptive insistence is a response to the wife’s steering of the interaction and the remark that he would probably “forget.”

(2) Husband (PA) interrupting wife (CP) twice (04:45)

01  CP °so I'll do the table° ((CP writes     Demonstrating understanding

02     answer)) (Fig 5a)

03     (0.9)

04  CP  cause you would probably (Fig 5b)

05     .hhh (0.6) forget [what

06  PA  [do the dishes             Interrupting; Steering

07     (0.3)

08  CP  uh no you ((touches PA's arm))        Disagreeing; Gesturing

09     [do =

10  PA  [you c'do the dishes            Disagreeing; Interrupting

11  CP  = the dishes [heh heh heh heh!

12  PA  [heh heh heh ((CP’s

13     body sways away from PA)) (Fig 5c)

Taking stock of the analysis so far, it is clear that there is a knot at the heart of scaffolding: It is generally effective, but sometimes fails; it is widely accepted, but often creates domination; it is requested but also often openly resisted. In order to explore these contradictory effects we introduce Charles Saunders Peirce’s theory of paradoxes.

According to Peirce [53] a sign, or representational meaning, arises out of a tripartite relation between an object, the sign, and an interpretant. The object is the thing or state of affairs being represented. The sign is the actual representation; either an icon, index or symbol. The interpretant is the semiotic system that interprets the relation between the sign and its object. Most other theories of meaning rest upon a binary between the sign and the signified [85]. Peirce’s theory is distinctive because signs do not signify anything except to an interpretant. Consequently, the meaning of any sign is as diverse as the interpretants that are brought to it.

Consider Excerpts 1 and 2. Arguably, what is being resisted in these excerpts is a shift from interpreting the interaction as progress in a joint activity to interpreting the interaction in terms of the PA’s disability. The utterances that refer to the PA as “a special case” and someone who will “forget” shift the focus from the task to the disability. Interpreting the interactions in terms of the PA’s disability is an ever-present option, but, it is not always salient. The question is, what actions and utterances might make this interpretant salient? While clearly, referring to the PA as a “special case” or likely to “forget” makes this interpretant salient, we also argue that the mere act of helping can index this interpretant.

Signs in context usually have multiple potential interpretants. Choosing to invite Bob for a meal might be interpreted as both Bob being a friend and living nearby. Choosing to cook chicken might be interpreted as both liking chicken and having experience cooking it. All these interpretations can co-exist without contradiction. However, sometimes the salient interpretants are contradictory, and then, Peirce argues, a paradox arises.

Peirce [86] considers the classic liar paradox, namely an utterance such as ‘this statement is false’ [87]. While normally such statements are considered meaningless, Peirce [86] argues that ‘far from being meaningless … it means two irreconcilable things’ [p.282]. First there is the interpretant relating to the propositional content. Second, there is the interpretant relating to the act of making a statement, the assumption being that what is said is intelligible and true. The paradox arises because the propositional content conflicts with the implicit assumption about intelligibility and truth. It follows that the paradox dissolves if one changes the second interpretant to: ‘What I say is un-intelligible and false.’ Thus while others have tried to solve the liar paradox in terms of hierarchical categories [88], Peirce solves the paradox using a theory of communication with multiple and conflicting interpretations. For example, statements such as ‘the apple is big and small’ are contradictory from the standpoint of classical logic, but, from Peirce’s point of view such statements are possible provided one acknowledges multiple interpretants (such as that of an ant and a human).

We propose that scaffolding may produce paradoxical effects, in exactly the sense described by Peirce. Consider the utterance ‘let me help you.’ The propositional content appeals to an interpretant that frames the activity as helpful. However, from the standpoint of what it means to receive help, the utterance could imply incompetence and dependency; in short, helplessness. This is different from referring to a PA as a “special case” or someone who will “forget” because those statements are not paradoxical; they do not appeal to contradictory interpretants. Helping activities, on the other hand, can be *simultaneously* empowering and disempowering.

**Fig 6:**
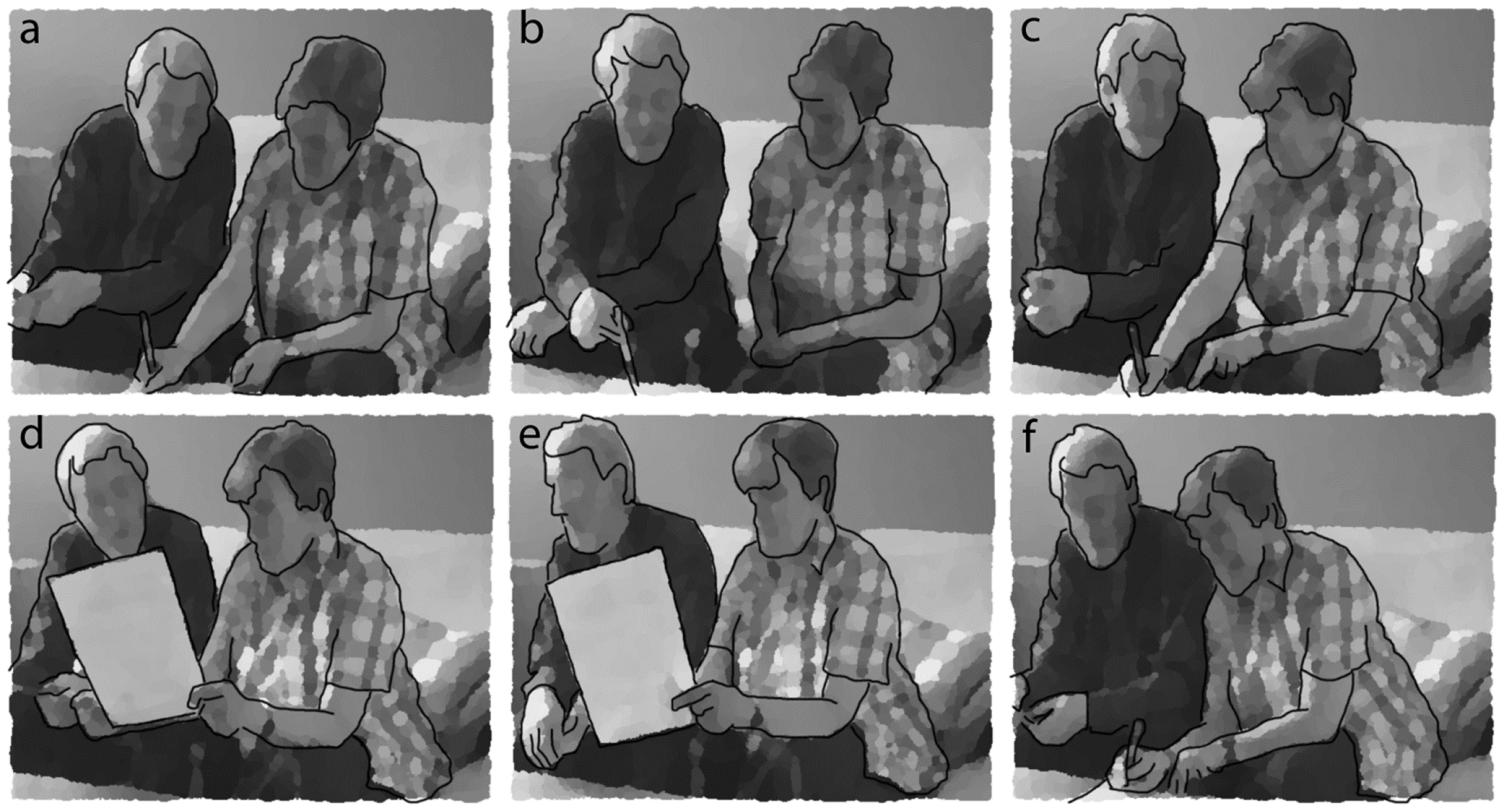
a-f. Anonymised video stills from excerpt 3 (CP is on left).

Excerpt 3 illustrates requesting and resisting help, and thus the paradoxical effects of scaffolding. It is from a married couple in which the wife has aphasia. The excerpt follows on from a sequence with ‘test’ questions [89] in which the husband had been teaching his wife how to spell “fish”–scaffolding that she resisted and then accepted. The excerpt begins with CP watching over PA’s shoulder as she is writing (Fig 6a) and then pointing to PA’s misspelling of “both” as “boht” with a pen (lines 01–02, Fig 6b). PA interrupts first with a glaring look and then, while shaking her head, an explicit resistance: “Don’t correct me … don’t want to be” (lines 04–07, Fig 6c). CP, ignoring this resistance, demonstrates the correct spelling and PA proceeds to correct her spelling with CP again looking over her shoulder. As she writes CP explains the mistake and praises her efforts (lines 12–13). PA, while accepting the help, simultaneously resists by saying “shhh!” (line 14). CP then reads the text aloud slowly, and this scaffolding is not requested or resisted (lines 18–25). However, when CP tries to solicit a response (lines 31–33), PA interrupts and resists, again insisting (“shhh”) on reading herself (line 32). But, then, in the subsequent turn (lines 34–35), PA gets stuck, and uses her pen to point to a word on the page, indicating a nonverbal request for CP to enunciate the word (lines 36–37, Fig 6d). CP obliges and confirms her comprehension (lines 39–40). PA continues reading, but gets stuck again, and nonverbally requests help once more (lines 47–49). This time, however, CP directs her to “just read” from the beginning (lines 54–55), and PA in an implicit act of resistance begins reading further on in the text. CP, evidently exasperated that his offer of help has been rejected again, looks away and drops his hands in a gesture of despondency (line 65, Fig 6e). PA then requests and receives confirmation of her comprehension of “gets” (lines 70–71) and “knocked o(h)ver” (lines 73–75). The excerpt ends with the couple reminiscing about a similar event that occurred before PA had a stroke, both are laughing (especially PA) and her body leans in to touch his (line 82, Fig 6f).

(3) Wife (PA) accepting, requesting and resisting help (11:06)

01  CP  right see what you've done again?      Correcting

02     ((points with pen)) (Fig 6b)        Gesturing

03     (1.1) [your writing

04  PA  [don't correct me            Interrupting

05  CP  hold on

06  PA  don't want to be ((shakes head;      Resisting help; Gesturing

07     points at question sheet)) (Fig 6c)

08  CP  °() ° ((CP takes question sheet

09     and writes; PA takes paper and

10     corrects own text; CP looks over

11     PA's shoulder as PA writes))

12  CP  °right yeh t- comes before the h-       Sounding-out

13     (.) same as the s- (.)

14  PA  shhh!                 Resisting help

15  CP  before the h- in fish°.h ((PA

16     glares at CP before continuing to

17     write))

18  CP  ((PA holds up paper and CP reads))

19     >once you're satisfied< (2.1)        Reading aloud

20     imagine that you ha:ve (1.3) almost

21     finished preparing (2.7) and your

22     friend or relative is expected to

23     arrive in ten minutes .hh (3.1) but

24     ↑then ((points with pen)) as you're

25     bringing the food (1.8) [it gets =

26  PA  [()                  Interrupting

27  CP  = knocked over

28     (4.3)

29  PA  °right° ((studies paper))          Demonstrating understanding

30     (0.8)

31  CP  right well [if Bob's arriving in =      Reformulating

32  PA  [() shhh                Interrupting; Resisting help

33  CP  = ten minutes what do you do?

34  PA  ((reads under breath to self))

35     °as you are° (7.2) () (3.7)

36     ((PA points to word on question       Requesting help; Gesturing

37     sheet for CP to see)) (Fig 6d)

38     (7.5)

39  CP  bring

40  PA  °bring° ((continues to read under

41     breath to self)) (1.0) ()

42  CP  °mhm°

43     (5.1)

44  CP  °um° ((CP points at page with pen))         Gesturing

45     (1.7)

46  PA  But ↑then ↑a:s (4.8) you (0.5)          Requesting help

47     ↑bring: (0.3) the food (12.4) I

48     don't know this one ((PA points         Gesturing

49     on question sheet))

50     (1.0)

51  CP  .hh

52     (0.3)

53  PA  [()

54  CP  [>just-just read it< (0.3) >from          Directing; Interrupting

55     but-< (0.3) >read just read<

56     (3.4)

57     ((PA points at question sheet          Requesting help; Gesturing

58     with pen))

59  CP  >read it out loud<

60     (1.6)

61  PA  but you started °() °              Disagreeing

62     ((PA points at question sheet;

63     PA begins reading later in the           Resisting help

64     sentence))

65  CP  hhhh ((looks away, drops hands))

66     (Fig 6e) (3.0) you can if you

67     [wanted to

68  PA  [()                     Interrupting

69  PA  ((PA reads to self)) it (0.4)           Reading aloud

70     gets (0.6)

71  CP  mhm

72     (0.3)

73  PA  knocked o(h)ver

74     (0.3)

75  CP  right                     Praising

76     (1.1)

77  PA  heh heh heh heh [heh heh!

78  CP  [remember what                  Interrupting

79     you did with [the turkey

80  PA  [heh heh! ((nods))

81  CP  that you dropped in the kitchen

82  PA  heh heh heh! ((nods)) (Fig 6f)

83  CP  that was [before you had a stroke

84  PA  [it was on the floor!                 Interrupting

Excerpt 3 demonstrates a knotted to-and-fro between help offered, help resisted, help accepted, and help requested. Help is offered several times, explicitly resisted thrice, accepted several times, and explicitly requested twice. This entanglement, we suggest, can be unpacked by distinguishing the interpretants.

Scaffolding, as a social action, is an indexical sign; an action that is meaningful from several standpoints, or interpretants. The act of scaffolding indexes, or implies, something about the recipient, the provider, and their relationship. One could interpret scaffolding as variably indexing: A focus and commitment to the task; care and concern for the recipient; the recipient’s lack of ability; the provider’s impatience; or, a relationship of dependency. Moreover, it might be that some scaffolding behaviours are more likely to index a certain foci (i.e., correcting is particularly focused on the recipient’s lack of ability, and thus perhaps, most likely to lead to resistance, as evident in excerpt 3). Nonetheless, all of these interpretants are available to all parties for all the scaffolding behaviours, but, there seems to be different emphasis placed on these interpretants by PAs and CPs.

Although both PAs and CPs generally focus on getting the task done, projecting positive identities, and sustaining the image of a normal life, PAs seemed more concerned with providing answers, rather than giving the correct answer, and moving the task forward, rather than getting hung up on small communication errors. CPs, on the other hand, seemed concerned to be good at facilitating communication (leading to solicitation) and at rehabilitation (leading to teaching sequences). These different orientations explain why PAs repeatedly wanted to move the task on, while CPs were repeatedly drawing attention to trivial communication errors.

The salient interpretants can also wax and wane during the course of an interaction. Specific interpretants come to the foreground when directly appealed to by interaction partners (such as referring to the PA as “a special case”). Also, interpretants based on the disability are likely to become salient when scaffolding fails, as it does about one third of the time (Table 1). Rather than indexing poor scaffolding (i.e., speaking too fast, inappropriate strategy) failure might index that despite receiving help the PA ‘still doesn’t get it.’ However, we want to argue, that these interpretants that frame the interaction in terms of disability are also indexed *by the act of helping itself*. This is the paradox of helping; the very act of helping, because it is a visible social act, indexes the need for help.

While previous research has shown that scaffolding risks positioning recipients as dependent and child-like [90], we have taken such analyses a step further by showing how helping is inherently paradoxical. Why do PAs simultaneously request and resist scaffolding? Because the effects of scaffolding are both positive and negative, simultaneously enabling communication within the relationship (evident in analysis 1) and creating an asymmetrical relationship (as revealed in analysis 2). Scaffolding, ostensibly an act of helping, is thus caught in a web of contradiction. It simultaneously facilities communication and indexes problems in communication; it simultaneously empowers and disempowers.

## Discussion: The paradox of helping

Across three analyses we have mapped out the range of scaffolding strategies evident in PA-CP conversations and examined their unintended effects. While scaffolding ostensibly improves communication, it can fail, creating a teaching-like genre of interaction, resulting in interaction dominance, and making salient disability.

Our first analysis builds on the literature that describes scaffolding strategies [91], especially in relation to communication [28]. Our analysis is distinctive because it examined both parties as equals within the scaffolding process, finding that PAs engage in all the scaffolding strategies observed, albeit usually with less frequency and considerably more variability. We also added to the literature by examining efficacy, finding that about a third of CP scaffolding initiatives fail. This latter finding, we suggest, is particularly important because scaffolding failures have the unintended consequence of making salient the communication disability.

Our second analysis builds on the literature that has documented the asymmetry in PA-CP conversations [26, 44], contributing a systematic analysis of the extent and type of interaction dominance. The observed asymmetry was mainly a result of CPs taking it upon themselves to solicit responses from the PA and to engage in teaching-like episodes–a genre of interaction far removed from the normative ideals of close social relationships, and, when combined with testing exchanges, identified as potentially problematic [89]. The concept of scaffolding originates in the field of child development [19, 92] where teaching-like episodes are less problematic. As the concept is broadened to apply to adults [4, 21], our results advise caution due to the unintended meanings of doing teaching episodes within close personal relationships.

Our third analysis identified a knot at the heart of scaffolding. On the one hand, scaffolding is widespread, usually accepted, and often requested, but, on the other hand, it can lead to asymmetries, resistance, and explicit rebuffs. To make sense of these tensions, we conceptualized helping as potentially paradoxical in Peirce’s [86] sense. Specifically, we suggested that CP scaffolding can have contradictory interpretations, simultaneously facilitating communication and marking the communication as problematic; simultaneously enabling and disabling. The literature on helping has found that people in diverse domains do not like being helped, specifically they worry that it will make them look incompetent [46]. Our introduction of Peirce and our examination of alternations between receiving, resisting, and requesting help show that helping is often a peculiarly paradoxical social activity. In trying to overcome a limitation it can make the limitation more salient.

The paradox of helping can be better understood if one conceptualizes helping as a gift. The literature on gifts shows that receiving a gift entails either obligation or subordination [1]. Accordingly, people will, as noted in the opening quotation from Mauss, often try to avoid receiving gifts [93], in much the same way as they resist requesting help. Of course the giver might not be interested in reciprocation; but, as with helping, there can be a divergence of perspective, and even if the feeling of obligation or subordination is one-sided it is still consequential. Arguably, a further complication with receiving help to communicate is that it often fails (Table 1), and one thing worse than being indebted for help received, is being indebted for help that was a hindrance.

Although the paradox of helping may be inherent in the semiotic structure of helping, conceptualizing helping as paradoxical in Peirce’s sense, does open some avenues for amelioration. First, scaffolding failure could be reduced. Scaffolding failure exacerbates the paradox of helping because it makes salient the help being provided and the problems that the disability is causing for the relationship. To this end, our findings support more research on training interventions for CPs [7, 10–13, 54]. We would also advocate the use of redundant scaffolding, such as gesturing while speaking and demonstrating understanding, because these strategies do not result in explicit failures.

Second, the quality of CP scaffolding could also be improved by giving PAs more control over the scaffolding they receive. PAs often blame comprehension problems on CPs speaking too fast or incorrectly supporting their comprehension [94]. We suggest developing a system of simple nonverbal signals that PAs (who have capacity for expression and gesture) could use to direct the scaffolding they receive. For example, gestures for ‘stop talking,’ ‘slow down,’ ‘rephrase,’ and ‘repeat’ would help the CP tailor their scaffolding to the needs of the recipient. This would facilitate the tailoring of strategies not just to the PA, but to the particular needs of the PA in a given context [17]. Moreover, such signals would have the significant secondary benefit of reducing CPs’ interaction dominance because PAs would be exerting more control within the conversation.

A third way to ameliorate the paradox of helping is to enable PAs to reciprocate the gift not yet repaid [1, 93]. Thus, ironically, what would help PA is if CP were to ask them for help, or, at least create opportunities for PA to repay help received. Although rare in our data, there were isolated instances, such as the PA who helped their CP spell “substitute.” Of course, the reciprocation does not need to be in the domain of communication; it could be in another domain which plays to the strengths of the PA. Relatedly, when PAs do engage in scaffolding, CPs should, perhaps, avoid resisting. Each of the 14 occasions when CPs resisted help was a missed opportunity for the PA to reciprocate.

Finally, the paradox of helping can, to some extent, be side-stepped, by raising the salience of alternative interpretants that focus on the quality of the CP-PA relationship. Given that receiving help with communication can be an identity threat, it is unsurprising to find that effective CPs focus on face-saving by redressing power imbalances, joking, and restoring dignity [10]. Eight of the 20 PAs never resisted help in part due to these subtle techniques. Particularly important, here, is humour leading to joint laughter. Laughter is a sequentially-organised activity that constructs intimacy [95] and is a common characteristic of informal caregiving relationships [2]. Laughter makes salient the interpretation that the relationship is enjoyable for both parties; pushing the interpretant of disability to the background. Mutual laughter indexes shared understanding, connectedness, symmetry, and enjoyment. However, humour itself can be asymmetrical, with CPs initiating most of the humour [96].

The current research has important limitations. The extent and types of aphasia among participants was diverse, as were their relationships. This likely resulted in the variable numbers of words spoken, variability in the frequency of strategy use and variability in the frequency of requesting and resisting scaffolding. Each dyad should be seen as having developed their own conversational style, with associated scaffolding strategies, through much trial and error experience [12–13, 17]. The limited size of our sample meant that we could not subset the data to systematically examine how types of relationship or aphasia profiles impact on scaffolding or interaction dominance. Another set of limitations concerns the task, which was ultimately artificial, and, with the presence of a Speech and Language Therapist, might have elicited genres of interaction associated with teaching and rehabilitation [89] (which is quite distinct from actual rehabilitation sequences [97]). Finally, the concept of scaffolding that we have used tends to assume an expert-novice relation with a power imbalance. This approach could be contrasted with research focused on conversation as a collaborative achievement [98]. By analysing the scaffolding done by both CPs and PAs, and systematically analysing initiation and responses, our research empirically documents the nature, extent and variability of the asymmetry.

There has been a lot of research on the scaffolding processes that CPs can use to facilitate communication with PAs. A neglected aspect of scaffolding, which we draw attention to, is the potential for multiple intended, unintended and contradictory effects. We have connected the literature on scaffolding with the literature on resisting help. While previous research has conceptualized resisting help in terms of identity threat, we used Pierce’s [86] semiotic approach to paradoxes to show that resisting help is a very peculiar communicative entanglement that can be conceptualized as a paradox; namely, an attempt to help, which is meant to empower, can simultaneously mark the recipient as powerless.

## Supporting information

S1 File This file contains the numeric data for the analyses of scaffolding strategies, interaction dominance, and resisting and requesting help. (XLSX)
